# First Complete Genome of Reticuloendotheliosis Virus in a Mallard Duck from Brazil: Phylogenetic Insights and Evolutionary Analysis

**DOI:** 10.3390/pathogens14020189

**Published:** 2025-02-13

**Authors:** Ruy D. Chacón, Claudete S. Astolfi-Ferreira, Stefhany Valdeiglesias Ichillumpa, Henrique Lage Hagemann, Maristela Furlan Rocha, Larissa Fernandes Magalhães, Tânia Freitas Raso, Antonio J. Piantino Ferreira

**Affiliations:** 1 Department of Pathology, School of Veterinary Medicine, University of São Paulo, Av. Prof. Orlando Marques de Paiva, 87, São Paulo 05508-900, Brazil; ruychaconv@alumni.usp.br (R.D.C.); csastolfi@gmail.com (C.S.A.-F.); henrique.trick@alumni.usp.br (H.L.H.); 2 Laboratorio de Fisiología Molecular, Instituto de Investigación en Ganadería y Biotecnología, Facultad de Ingeniería Zootecnista, Agronegocios y Biotecnología, Universidad Nacional Toribio Rodríguez de Mendoza de Amazonas, Chachapoyas 01001, Peru; stefhanyvaldeiglesias@gmail.com; 3 Clínica de Aves, Rua Voluntario Mario Mazini, 1697, São Paulo 14405-094, Brazil; maristelafurlanrocha56@gmail.com (M.F.R.); larissafmagalhaes@yahoo.com (L.F.M.)

**Keywords:** *Gammaretrovirus*, reticuloendotheliosis virus, genome, mallard duck, molecular characterization, phylogenetic tree, selective pressure

## Abstract

Reticuloendotheliosis virus (REV) is an oncogenic retrovirus that affects both commercial and free-ranging birds. To date, only two complete REV genome sequences have been identified in chickens from South America, with no records in other avian species. This study reports the first complete genome of REV detected in a mallard duck (*Anas platyrhynchos domesticus*) in South America. In 2021, a mallard duck in Brazil died from severe lymphoproliferative disease affecting multiple organs. Molecular detection and histopathological analysis confirmed REV as the causative agent. Using dideoxy sequencing and phylogenetic analysis, the virus was classified as subtype 3 (REV-3). The phylogenetic analysis identified three clades, each with a bootstrap value of 100, corresponding to the three REV subtypes. Furthermore, a comprehensive comparative genomic analysis revealed two distinct REV-3 subclusters—‘East’ (38 strains) and ‘West’ (24 strains)—with notable geographical associations. Additionally, 27 genomes in chimeric states with fowlpox virus (FWPV) were distributed across the phylogenetic tree, emphasizing the critical role of FWPV in the dissemination of REV. Selective pressure analysis revealed evidence of positive selection acting on several codons within the *gag*, *pol*, and *env* genes, particularly in domains such as matrix, p18, reverse transcriptase/ribonuclease H, and surface. These findings provide valuable insights into REV evolution and underscore the importance of genomic surveillance for detecting REV circulation in diverse hosts.

## 1. Introduction

Reticuloendotheliosis virus (REV) is a retrovirus that infects a wide range of avian species, including both wild birds and poultry. It belongs to the *Gammaretrovirus* genus and is associated with various clinical syndromes, primarily chronic and acute neoplasias, immunosuppression, and subclinical or asymptomatic infections [1].

REV viral particles are spherical or slightly pleomorphic, measuring approximately 100 nm in diameter, and are enclosed by a lipid envelope with associated surface glycoproteins. Its genome consists of linear, positive-sense RNA, which is converted into double-stranded DNA during the proviral state [1]. The REV genome is 8.2 to 8.4 kb in length and contains the *gag*, *pol*, and *env* genes, flanked by identical long terminal repeat (LTR) sequences. As a retrovirus, REV integrates into the genome of host cells. Notably, REV can also integrate into the genomes of other DNA viruses, forming viral chimeras, primarily with poxviruses and herpesviruses [2,3]. These chimeric integrations or co-infections can lead to exacerbated or atypical clinical conditions [4,5] and may compromise vaccine efficacy [6,7].

Transmission of REV occurs through various routes. Vertically, the virus can be transmitted via semen from birds with persistent viremia. Horizontally, it spreads through close contact with feces, body fluids, or contaminated litter [1]. Additionally, hematophagous insects and arachnids can transmit REV after feeding on infected blood, and mechanical transmission by insects has also been documented [1,8,9]. The dissemination of chimerized viruses, such as the fowlpox virus (FWPV), further complicates control efforts due to the wide range of free-living hosts [5,10]. Cases of REV outbreaks linked to contaminated vaccines underscore the need for stringent vaccine quality control [11].

In the poultry industry, REV has a significant economic impact, as infections lead to reduced productivity and increased susceptibility to coinfections [1]. While monitoring in commercial settings is well-structured, detecting REV in free-living birds presents greater challenges. Reports are often limited to specific surveillance programs or isolated cases when clinical signs become evident, or when populations experience increased mortality [12,13,14].

Most REV studies focus on poultry, particularly chickens [15,16,17]. Reports on waterfowl poultry are scarce, but some have documented increased mortality accompanied by lymphomas in visceral organs, such as in *Anser anser* [18] and *Cairina moschata* [19]. Similarly, in wild waterfowl, REV has been associated with morbidity and mortality in *Branta sandvicensis* and *Phoebastria immutabilis* in Hawaii, USA [14].

REV has been reported in numerous countries, infecting both poultry and free-living birds, either alone or in coinfection with other pathogens [12,13,14,16,20,21]. However, in South America, REV has been scarcely studied. Apart from Brazil, only serological reports exist in other countries, showing positivity rates ranging from 33.3% in Peru [22] to 95% in Argentina [23]. In Brazil, REV has been documented in commercial birds [5,17], backyard birds [24], and free-living birds [25]. However, only two complete genomes have been sequenced from commercial birds in the country.

This study aims to report and characterize the first complete genome of reticuloendotheliosis virus (REV) detected in an *Anseriform* species in Brazil. Additionally, through comparative genomics and selective pressure analysis, it seeks to update the current understanding of sequenced REV strains, providing insights into their evolution and global geographic distribution.

## 2. Materials and Methods

### 2.1. Clinical Case

In April 2021, a mallard duck (*Anas platyrhynchos domesticus*) was treated at a veterinary clinic in Franca, São Paulo, Brazil. The 4-month-old female bird was brought in for clinical evaluation due to a noticeable increase in cervical volume. The duck was free-range and cohabitated with a small group of backyard chickens. The owner reported that 16 months earlier, a hen had died after exhibiting signs of apathy and anemia.

Initial clinical examinations included a complete blood count, radiography, and fine-needle aspiration biopsy (FNAB). The blood count revealed normocytosis, monochromia, irregular atypical lymphocytes, and heterophils with marked toxic granulation. Radiographic imaging of the cervical region showed an area of increased volume with well-defined, regular contours and soft-tissue radiopacity in the caudal cervical region. This mass extended from the direct lateral aspect to the ventral portion, causing lateral displacement of the trachea to the left and compression of the cervical air sacs. The tracheal lumen and cervical vertebrae appeared unaltered.

An FNAB of the cervical nodule revealed mitotic figures in lymphocytes, leading to a preliminary diagnosis of lymphoma. Based on these findings from the clinical examination and anamnesis, an oncogenic virus was suspected as a potential etiological agent. The duck died the following day and was submitted for necropsy, histopathological examination, and molecular analysis. Sample examination and handling were conducted using Personal Protective Equipment (PPE) (Delta Plus Brasil, São Paulo, Brazil) and sterile laboratory gloves. Histopathological evaluation was also performed on the liver and spleen of the hen that had died months earlier at the same location.

### 2.2. Molecular Detection of Oncogenic Viruses and Histopathology

For molecular analyses, liver and blood samples were processed. The liver tissue was macerated under sterile conditions, and both liver and blood samples were mixed with phosphate-buffered saline (PBS) in a 1:1 volume ratio. The samples underwent three cycles of freezing (−80 °C for 10 min) and thawing (56 °C for 1 min). Following centrifugation at 12,000× *g* for 20 min, 200 µL of the supernatant was collected for nucleic acid extraction. Samples that tested positive for oncogenic viruses in a previous study were used as positive controls [24], while PBS was used as negative control.

DNA was extracted using the MagMAX™ Viral/Pathogen Nucleic Acid Isolation Kit (Applied Biosystems, Austin, TX, USA), and its concentration was measured with a NanoDrop 2000 spectrophotometer (Thermo Fisher Scientific, Wilmington, DE, USA). Molecular detection of oncogenic viruses was performed using multiplex PCR as described by Gopal et al. (2012) [26]. This assay simultaneously detects avian leukosis virus (ALV), Marek’s disease virus (MDV), and reticuloendotheliosis virus (REV). The amplification targets include the *p27* gene for ALV (primers F: CCCGATYACTATGGCRGAAG and R: CRGCTATGCCTYGATCCGTA), the *meq* gene for MDV (primers F: CTGACGGCCTATCTGAGGAG and R: GGAAACCACCAGACCGTAGA), and the LTR region for REV (primers F: TGAGGGAAAATGTCATGYAAC and R: ATCCCTACCCCACCCAGTAGG). PCR conditions include an initial denaturation at 94 °C for 5 min; a cycling phase including denaturation at 94 °C, annealing at 55 °C, and extension at 72 °C, for 45 s at each temperature; and a final extension phase at 72 °C for 7 min. Visualization of the PCR products was performed by electrophoresis in a 1.5% agarose gel.

For histopathological examination, samples from the cervical nodule, liver, pancreas, bursa, kidney, ovary, and skin were fixed in 10% formalin and embedded in paraffin. Sections measuring 5 µm were prepared using a microtome, stained with hematoxylin and eosin (HE), and observed under conventional light microscopy.

### 2.3. Complete Genome Sequencing of Reticuloendotheliosis Virus

The complete genome of REV was obtained using Sanger sequencing (dideoxy sequencing), following the methodology described in a previous study [17]. This approach involves amplifying 13 overlapping fragments that cover the entire REV genome, including both long terminal repeats (LTRs) as well as the *gag*, *pol*, and *env* genes. A sample from the pGEM^®^-T Vector System (Promega, Madison, WI, USA) was used as a sequencing validation control. The resulting electropherograms were trimmed and assembled using Geneious Prime^®^ 2020.2.4 software.

The assembled genome has been deposited in GenBank under the accession number PQ186185.

### 2.4. Sequence and Phylogenetic Analysis

Sequence analyses were conducted using the genome assembled in this study alongside 70 complete coding sequences (CDSs) of REV, including those integrated into FWPV genomes, available in GenBank. The sequences were aligned using the software MAFFT v7 [27], and the identity matrix of nucleotides was produced. The optimal nucleotide substitution model was determined with ModelTest-NG v0.1.7 due to its better performance and time in whole genome alignments [28]. A phylogenetic tree was constructed using the maximum likelihood method implemented in the RAxML program v. 1.2.2 [29], with the following parameters: start tree(s): random (10) + parsimony (10), random seed: 1733389877, pattern compression: ON, site repeats: ON, logLH epsilon: general: 10.000000, brlen-triplet: 1000.000000, fast spr radius: AUTO, spa subtree cutoff: 1.000000, fast CLV updates: ON, branch lengths: proportional (ML estimate, algorithm: NR-FAST), SIMD kernels: AVX2. The final tree was subsequently edited using iTOL v6 [30].

### 2.5. Selective Pressure

Selective pressure analysis was performed on the coding regions of the 70 complete REV genomes. To identify pervasive positive selective pressure, the FUBAR method (Prob [α < β] > 0.9), SLAC method (P [dN/dS > 1] < 0.1), and FEL method (*p*-value < 0.1) were applied. To detect episodic positive selective pressure, the MEME method was used with a *p*-value threshold of <0.1. All analyses were conducted using the Datamonkey platform (https://datamonkey.org/ accessed on 12 November 2024) [31]. The sites under positive selective pressure were mapped onto each domain of REV proteins using IBS v1.0 [32].

## 3. Results

### 3.1. Necropsy, Molecular Detection of Oncogenic Viruses and Histopathology

**Figure 1:**
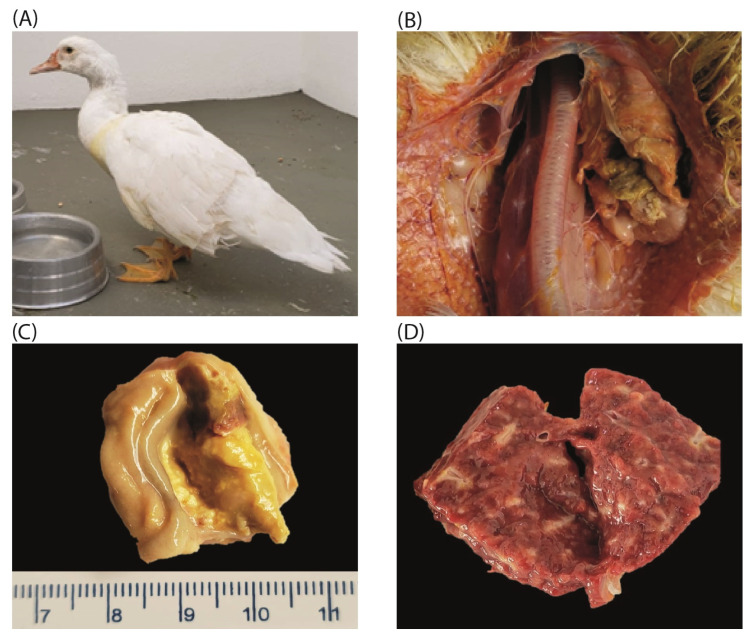
Necropsy findings in the mallard duck. (**A**) Unilateral cervical nodule enlargement. (**B**) Enlargement of the thymus. (**C**) Enlargement of the Bursa of Fabricius. (**D**) Multifocal pale areas on the liver surface with a marble-like appearance.

During the necropsy, a unilateral cervical nodule was identified in the thymus region, corresponding to the area observed externally (Figure 1A). Additional findings included thymic enlargement (Figure 1B) and enlargement of the Bursa of Fabricius (Figure 1C). The liver exhibited hepatomegaly with multifocal pale areas, giving the surface a marble-like appearance (Figure 1D). The spleen was friable and hemorrhagic, while the kidneys displayed pale areas on their surfaces.

Molecular detection using multiplex PCR confirmed the presence of REV in both liver and blood samples. No evidence of ALV or MDV was detected.

**Figure 2:**
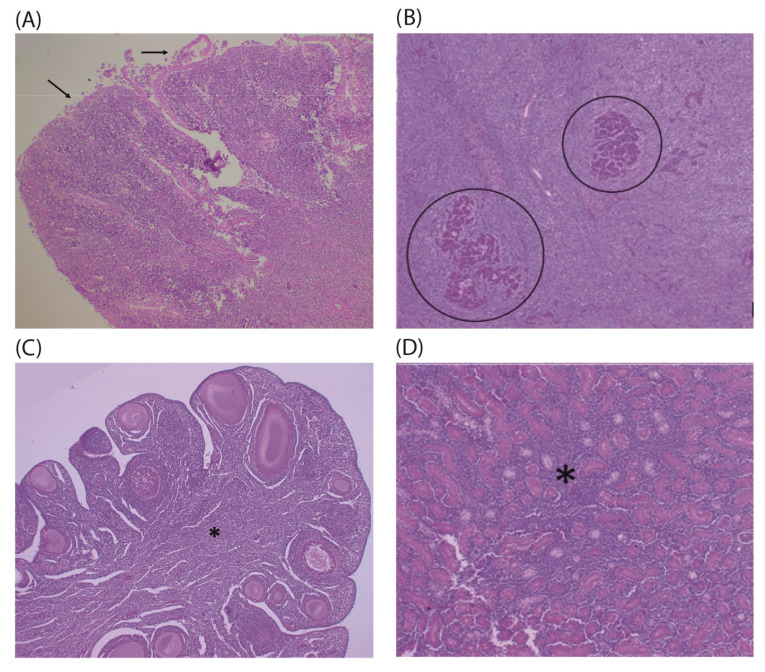
Lymphomas in the mallard duck. (**A**) The Bursa of Fabricius shows areas of ulceration and epithelial detachment (arrow), with diffuse proliferation of lymphoblasts arranged in a mantle. (**B**) Proliferative infiltrates of lymphoblasts widely expand and replace the liver parenchyma with remnants of hepatocyte cords (circle). (**C**) The ovary exhibits discrete primary oocytes infiltrated by lymphoblasts, with the medullary region diffusely affected by neoplasia (asterisk). (**D**) The kidney shows lymphoblasts promoting expansion of the interstitium (asterisk). Hematoxylin and eosin staining (HE).

Histopathological analysis of the mallard duck revealed the proliferation of monomorphic lymphoblasts arranged in a mantle and anchored in discrete fibrovascular stroma, observed in several organs, including the cervical node, liver, pancreas, bursa, kidney, ovary, and skin (Figure 2 and Figure S1). The cells exhibited a high nucleus-to-cytoplasm ratio, with minimal cytoplasm, and large nuclei with nucleoli, which ranged from single to multiple (Figure S2). In the cervical mass, bursa, pancreas, and ovary, the proliferation was diffuse, while in the kidneys and liver, it was multifocal to coalescent. In the skin, only discrete foci of neoplastic cells were observed in the dermis.

It is important to note that an aspiration biopsy was initially performed on the cervical mass, followed by collection of the nodule at necropsy. In both cases, the microscopic features were similar, with diffuse proliferation of lymphoblasts and the absence of tissue reference, making it impossible to affirm the organ. In the necropsy sample, collected 15 days after the biopsy, there was evidence of extensive necrosis (approximately 90%) in the sampled fragments, indicating rapid progression and a poor prognosis (Figure S1).

Interestingly, retrospective histopathological analysis of liver and spleen samples from a hen that had died at the same location 16 months earlier revealed similar findings. In the liver, there was a loss of tissue architecture with extensive coalescent areas of neoplastic lymphocyte proliferation, monomorphic lymphoblasts, and frequent mitotic figures (Figure S3). In the spleen, there was nodular to diffuse proliferation of uniform lymphoid cells with a loss of splenic architecture (Figure S4).

### 3.2. Complete Genome Sequencing and Phylogenetic Analysis

The complete proviral genome of strain USP-2094 was obtained after assembling 26 electropherograms, achieving 93.1% quality per site >Q30, with an average electropherogram length of 525.8 bp, Std Dev: 272.9, and Error-Free Odds of <0.0001% after trimming. The genome is 8284 base pairs (bp) long, with a GC content of 52.3%. The Long Terminal Repeats (LTRs) are identical as expected, spanning positions 1 to 543 (5′ end) and 7742 to 8284 (3′ end). The genes extend from 934 to 2433 (*gag*), 2434 to 6015 (*pol*), and 5952 to 7712 (*env*), which overlap with the *pol* gene. These genes encode proteins of 499, 1193, and 586 amino acids, respectively.

After aligning the REV coding genomes, a sequence identity matrix was generated (Table S1). Strain USP-2094 showed 97.95% identity with strain HA9901, the REV-1 subtype reference, and 96.64% identity with the SNV strain, the REV-2 subtype reference. Comparisons with REV-3 subtype strains revealed a sequence identity ranging from 98.17% to 99.94%.

**Figure 3:**
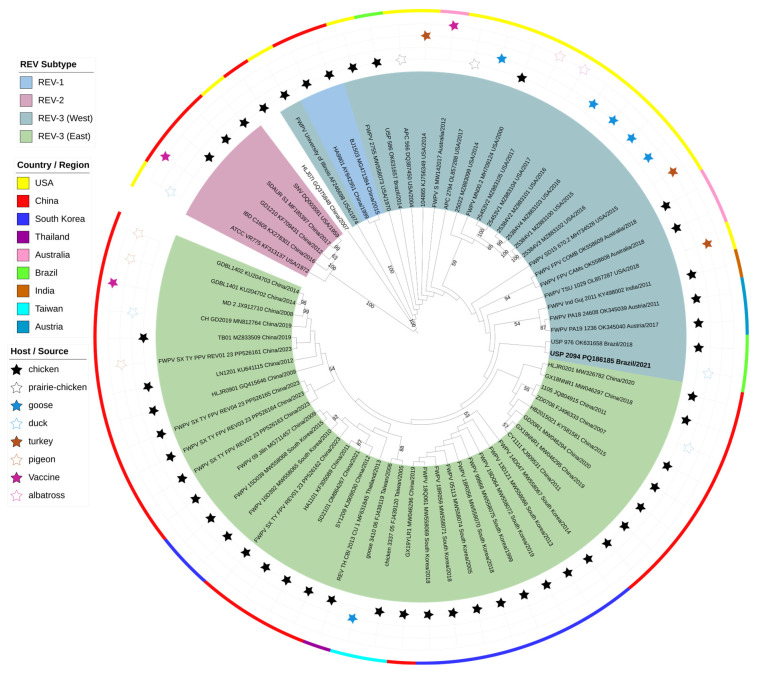
A maximum-likelihood phylogenetic tree was constructed using 70 complete coding sequence genomes, including the USP-2094 strain from this study. The tree was inferred with the TPM3uf+G4 substitution model, and support values based on 1000 bootstrap replicates are shown on the branches. Clades corresponding to REV subtypes are color-coded. Additional annotations include the country of detection (indicated by a colored ring) and the host/source of each strain. The host/source of each genome is indicated by stars, categorized as follows: chicken (filled black star), prairie-chicken (empty black star), goose (filled blue star), duck (empty blue star), turkey (filled orange star), pigeon (empty orange star), vaccine (filled pink star), and albatross (empty pink star). The MDV strain from this study is highlighted in bold.

The alignment of the coding genome spans 6782 base pairs. ModelTest-NG identified TPM3uf+G4 as the most suitable nucleotide substitution model. Phylogenetic analysis revealed three subtypes of REV (Figure 3). The REV-3 subtype was the most abundant and distinguished two major clusters. The “East” cluster contained the largest number of strains, all exclusively of Asian origin, including those from China, South Korea, Thailand, and Taiwan. The “West” cluster included strains from various continents and countries, such as the USA, Brazil, Austria, Australia, and India. The strain sequenced in this study (USP-2094) was grouped in the West cluster, as expected. The closest strain was USP-976, also from Brazil, isolated from a chicken with proventriculitis in 2018 [17].

The genomes analyzed originated from various hosts, primarily chickens. However, the phylogenetic grouping was mainly based on geographic origin, as shown by the distinct clusters representing Asia, the USA, and Brazil, including strains from different hosts. Additionally, this study included all available genomes of REV chimerized with the fowlpox virus (denoted by the acronym FWPV at the beginning of the strain name). As shown in Figure 3, these genomes are clustered closely together. Interestingly, FWPV-REV genomes from the West, including those from the USA, Australia, India, and Austria (collected between 2011 and 2018 from chickens and turkeys), were notably close to each other. Furthermore, the oldest strains (FWPV University of Illinois from 1974 and FWPV 2755 from 1970) were positioned more basally, suggesting that FWPV has significantly contributed to the spread of REV.

### 3.3. Selective Pressure

The selective pressure analysis performed on the alignments of coding regions enabled the identification of probable signals from codons subjected to evolutionary forces. Among these, diversifying forces or positive selection stand out, which can be pervasive (acting throughout the evolutionary tree) or episodic (acting only on some lineages).

**Figure 4:**
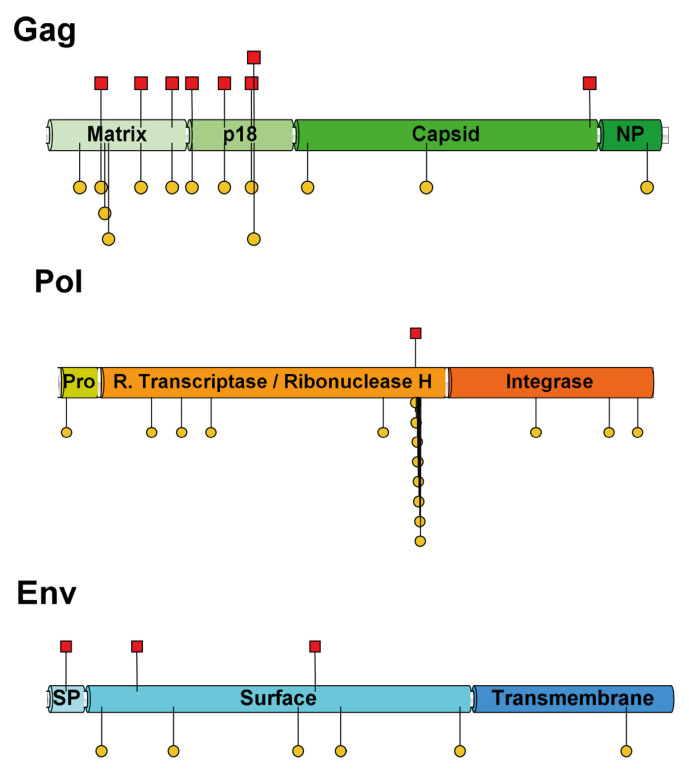
Schematic representation of REV proteins, highlighting their domains and sites with positive selective pressure. NP: nucleoprotein. SP: signal peptide. Codons under positive selection are represented as red-filled squares (pervasive) or yellow-filled circles (episodic).

In the *gag* gene, 14 codons were identified as exhibiting positive selective pressure (either pervasive and/or episodic) (Table 1). Thirteen of these codons showed evidence of episodic positive pressure, while eight displayed pervasive positive pressure. These codons were distributed across the four domains of *gag*: matrix, p18, capsid, and nucleoprotein. Interestingly, 10 of the 14 codons (71.43%) are located in the first third of the *gag* gene, which includes the matrix proteins and p18 (Figure 4).

In the *pol* gene, 16 codons exhibited signals of episodic positive pressure, with 1 codon (codon 691) also showing evidence of pervasive positive pressure. These codons were distributed across the three main domains: pro-pol, reverse transcriptase/ribonuclease H, and integrase. Notably, 12 of the 16 codons (75%) were located in the reverse transcriptase/ribonuclease H domain (Figure 4). Additionally, the region comprising codons 691 to 700 showed selective pressure signals at eight sites. Deletions were observed in three Chinese strains within this region.

In the *env* gene, nine codons were found to exhibit positive selective pressure signals (either pervasive and/or episodic). Six of these showed episodic positive pressure, while the remaining three exhibited pervasive positive pressure (Figure 4). These sites are distributed across the three domains of *env*: signal peptide, surface, and transmembrane. However, seven of the nine sites (77.78%) were located in the surface domain.

## 4. Discussion

REV is an oncogenic and immunosuppressive virus capable of infecting various species across different avian orders. The available complete REV genomes are mostly of chicken origin. This study sequenced the genome of a REV strain detected in a backyard mallard duck (*Anas platyrhynchos domesticus*) in Brazil.

REV is associated with various clinical manifestations in birds, including runting disease syndrome, abnormal feathering, paralysis, and increased mortality. When birds develop chronic lymphomas or acute reticulum cell neoplasms, they often show few obvious clinical signs due to the rapid progression of the disease, which typically leads to death [1]. In ducks and geese, primarily spontaneous forms of the disease have been reported since the early studies, often associated with tumor formation in various organs [18,19,33,34]. This study aligns with these spontaneous clinical manifestations, showing rapid progression with generalized neoplasms in multiple organs that ultimately led to the death of the infected animal. However, unlike the aforementioned studies, this case occurred on a free-range family farm.

At the histopathological level, the biopsy and necropsy results support the rapid progression and poor prognosis of the disease, with extensive areas of necrosis [14,19]. Cellular damage is widespread across various tissues. As seen in this study, proliferative infiltrates of lymphoblasts and lymphocytes commonly affect cellular architecture [14,18,19]. Additionally, nuclear-to-cytoplasm ratio discrepancies, irregular nuclei, and the presence of mitotic figures have also been reported [18,35].

An intriguing finding of this study was the histopathological evidence suggesting the persistence or circulation of REV in the same geographical area for at least 16 months. Several factors may have contributed to this scenario, such as horizontal transmission mediated by mechanical or hematophagous vectors [1,8,9], or persistence facilitated by environmentally resistant carrier viruses like MDV and FWPV [2,3]. Additionally, interspecies transmission cannot be ruled out. However, further studies are required to confirm and validate this possibility.

Genomic analysis of REV has consistently shown a fairly conserved genome and the presence of at least three major subtypes. Subtype 1 (REV-1) is the smallest cluster, including the reference strain HA9901, isolated from chickens in China in 1999 [36]. Subtype 2 (REV-2) includes the prototype SNV strain isolated from chickens in the USA in 1959 (initially called spleen necrosis virus) and the DIAV strain isolated from ducks in the USA in 1972 (initially called duck infectious anemia virus) [37]. Subtype 3 (REV-3) represents the largest cluster, including strains from the 1970s detected in co-infection with fowlpox virus (FWPV 2755, FWPV University of Illinois) [38]. This subtype also encompasses strains from diverse temporal, geographic, and host origins. Our sequenced strain, USP-2094, is located in this REV-3 subtype, consistent with all studies that include multiple genomes in their analysis [11,13,15,16,17,18,19,35,39]. However, this study took an exhaustive and comprehensive approach, incorporating all available complete genomes, including those integrated into FWPV genomes, totaling 70 strains. As a result, in addition to the expected clustering into three subtypes, this study identified, for the first time, two major subclusters within REV-3. One subcluster, named “East,” includes only Asian strains isolated between 2005 and 2023 from different hosts, both in the free state and chimerized with FWPV. The other REV-3 subcluster, named “West”, mainly includes strains from Western countries and continents detected between 1970 and 2021 (including our USP-2094 strain). The basal positioning of these strains suggests a common origin for REV-3, which then dispersed to different regions. However, in Asia, strains derived from this lineage exhibit a higher degree of genetic similarity, indicating homogeneous selective pressure or lower evolutionary variability in this region. This subcluster also includes strains from various hosts and genomic states of REV.

A notable characteristic of REV is its ability to chimerize with viruses that have long dsDNA genomes, such as MDV and, predominantly, FWPV [2,3]. In the case of MDV, various studies have reported coinfection with REV [16,24,39]. Additionally, it has been shown that coinfection with REV increases pathogenicity and reduces vaccine efficacy [7,24,40]. For FWPV, reports are even more abundant [5,10,20,21,41,42,43]. The region between ORFs 201 and 203 of the FWPV genome has been identified as an integration hotspot, with two main states observed: integration of nearly the complete REV genome and/or only the LTRs [3,5,10,38,44]. Similar to MDV, coinfection with REV in FWPV has been associated with increased severity, immune evasion, and even atypical symptoms [4,5,21,38,44]. This study analyzed 27 nearly complete REV genomes integrated into FWPV, revealing their wide distribution within the REV-3 subtype, including both the East and West clusters. Remarkably, this phenomenon has been observed throughout the period covered by REV-3 strains (1970–2023), suggesting its significant role in the virus’s dispersion. Furthermore, although this phenomenon has primarily been detected in chickens and domestic turkeys [5,10,21], its detection in pigeons and wild turkeys highlights the potential for transmission in free-living birds as well [43,45].

Selective pressure is a key evolutionary mechanism shaping the genetic diversity of avian oncogenic viruses [46,47,48,49]. The analyses conducted in this study identified, for the first time, several REV codons potentially influenced by selective pressure, highlighting the virus’s adaptive evolution. However, the distribution and concentration of these sites are not uniform, reflecting the differential accumulation of nonsynonymous mutations across functional genomic regions. In the *gag* gene, matrix proteins and p18 exhibit a significantly higher accumulation of sites under pervasive positive pressure. This pattern suggests that these proteins may be critical targets of host immune responses or play key roles in viral assembly and release, as seen in other retroviruses [50,51]. The *pol* gene, while more conserved, shows an accumulation of episodic positive pressure codons in the reverse transcriptase/ribonuclease H domain, particularly in a region (codons 691–700) characterized by mutations and deletions [14,43]. Given that reverse transcriptase is essential for viral replication, these selective pressures likely reflect functional adaptations that enhance replication efficiency, mutational robustness, or resistance to host restriction factors, consistent with observations in other retroviruses [14,52]. In the *env* gene, positive selection is more pronounced within the surface domain, which contains the receptor-binding domain (RBD). Mutations in this region can directly influence the virus’s ability to engage host cell receptors, potentially modulating tropism, immune evasion, or transmission efficiency [53,54,55]. These findings align with the broader understanding of retroviral evolution, where surface glycoproteins are frequent targets of selective pressure due to their role in host–virus interactions. Overall, the patterns of selective pressure observed in REV suggest a dynamic evolutionary landscape shaped by host–virus interactions and functional constraints on viral proteins.

This study expanded the genomic data on REV in Brazil, particularly in a mallard duck. The presence of REV in waterfowl cohabiting with *Galliformes* suggests a risk of cross-species transmission, posing potential threats to both poultry production and wildlife conservation, as observed with other avian viruses [56,57]. Furthermore, the circulation and persistence of immunosuppressive viruses in domestic animals pose a potential risk to both animal and public health, as they facilitate and prolong infections caused by other devastating pathogens [58]. These findings emphasize the need for enhanced surveillance strategies and biosecurity measures, with broader inclusion of species that may serve as reservoirs, vectors, or directly affected hosts.

However, this study has some limitations. As an isolated clinical case, the sequenced genome may not fully represent the genetic diversity of REV strains circulating in the region. Additionally, while our findings suggest potential transmission between *Galliformes* and *Anseriformes*, this study could not determine whether such transmission is imminent, direct, or mediated by vectors.

Overall, these results highlight the urgent need to expand genomic surveillance of REV in avian populations across Brazil and other South American countries. Such efforts will provide a deeper understanding of REV’s evolutionary history and help identify potential host-specific adaptations. On the other hand, monitoring waterfowl provides insights into the health status of both residents and migratory species, emphasizing the importance of establishing regional networks or collaborative efforts.

## 5. Conclusions

The complete genome sequenced in this study contributes to advancing the understanding of REV in diverse hosts. Additionally, the comprehensive phylogenetic analysis identified subclusters within the REV-3 subtype, shedding light on its geographic dispersion. The widespread presence of FWPV across various regions and time periods underscores its significant role in the dissemination and persistence of REV. Furthermore, the detection of codons under selective pressure in key REV genes and protein domains highlights their potential involvement in critical functions such as virion release, adaptation, and receptor binding. Continued genomic surveillance and future studies will be essential for unraveling the evolutionary and dissemination mechanisms of REV.

## Figures and Tables

**Table 1:** Sites under pervasive and episodic positive selection pressure in REV coding genes.

Gene	Codon Position	FUBAR ^1^ Probability α < β	FEL ^2^ *p*-Value	MEME ^2^ *p*-Value
*gag*	28	– ^3^	–	0.085
	45	0.987	0.0458	0.064
	48	–	–	0.08
	51	–	–	0.038
	77	0.968	0.0886	0.005
	102	0.945	–	0.047
	118	0.925	–	0.032
	144	0.914	–	0.047
	166	0.903	–	0.03
	168	0.943	–	0.075
	211	–	–	0.063
	306	–	–	0.1
	437	0.91	–	–
	483	–	–	0.094
*pol*	15	–	–	0.077
	180	–	–	0.085
	238	–	–	0.006
	295	–	–	0.083
	628	–	–	0.041
	691	0.926	0.0669	0.001
	692	–	–	0
	694	–	–	0.005
	695	–	–	0.018
	696	–	–	0.093
	697	–	–	0.004
	699	–	–	0.011
	700	–	–	0.001
	924	–	–	0.024
	1066	–	–	0.085
	1121	–	–	0.016
*env*	18	0.902	–	–
	51	–	–	0.003
	84	0.957	–	–
	118	–	–	0.076
	234	–	–	0.078
	250	0.910	–	–
	274	–	–	0.094
	385	–	–	0.058
	540	–	–	0.042

^1^ FUBAR (*p* > 0.9). ^2^ MEME, FEL, SLA C (*p* < 0.1). ^3^ –: no signal detected.

## Data Availability

The assembled genome has been deposited in GenBank under the accession number PQ186185.

## Supplementary Materials

The following supporting information can be downloaded at: https://www.mdpi.com/article/10.3390/pathogens14020189/s1, Figure S1: Lymphoma in the cervical nodule of the mallard duck; Figure S2: Lymphoma in the mallard duck; Figure S3: Lymphoma in the liver of chicken; Figure S4: Lymphoma in the spleen of chicken; Table S1. Identity matrix of nucleotide sequences from the complete coding genome sequences of REV strains used in this study.
