# Supplementary material for: First Complete Genome of Reticuloendotheliosis Virus in a Mallard Duck from Brazil: Phylogenetic Insights and Evolutionary Analysis
Source: Pathogens. 2025 Feb 13;14(2):189. doi: 10.3390/pathogens14020189 (PMC11858360; doi:10.3390/pathogens14020189)
Supplement: Supplementary file 1 [file pathogens-14-00189-s001.zip › Supplementary Figures.pdf]

pathogens

MDPI

Supplementary Materials

First Complete Genome of Reticuloendotheliosis Virus (REV) in a Mallard Duck from Brazil: Phylogenetic Insights and Evolutionary Analysis

Ruy D. Chacón [1], Claudete S. Astolfi-Ferreira [1], Stefhany Valdeiglesias Ichillumpa [2], Henrique Lage Hagemann [1], Maristela Furlan Rocha [3], Larissa Fernandes Magalhães [3], Tânia Freitas Raso [1,*], and Antonio J. Piantino Ferreira [1,*]

[1] Department of Pathology, School of Veterinary Medicine, University of São Paulo, Av. Prof. Orlando Marques de Paiva, 87, São Paulo 05508-900, Brazil; ruychaconv@alumni.usp.br (R.D.C.); csastolfi@gmail.com (C.S.A.-F.); henrique.trick@alumni.usp.br (H.L.H.)

[2] Laboratorio de Fisiología Molecular, Instituto de Investigación en Ganadería y Biotecnología, Facultad de Ingeniería Zootecnista, Agronegocios y Biotecnología, Universidad Nacional Toribio Rodríguez de Mendoza de Amazonas, Chachapoyas, Peru; stefhanyvaldeiglesias@gmail.com (S.V.I.)

[3] Clínica de Aves, Rua Voluntario Mario Mazini, 1697, São Paulo 14405-094, Brazil; maristelafurlanrocha56@gmail.com (M.F.R.); larissafmagalhaes@yahoo.com (L.F.M.)

* Correspondence: tfraso@usp.br (T.F.R.); Tel.: +55-11-3091- 1230; ajpferr@usp.br (A.J.P.F.); Tel.: +55-11-3091-1352

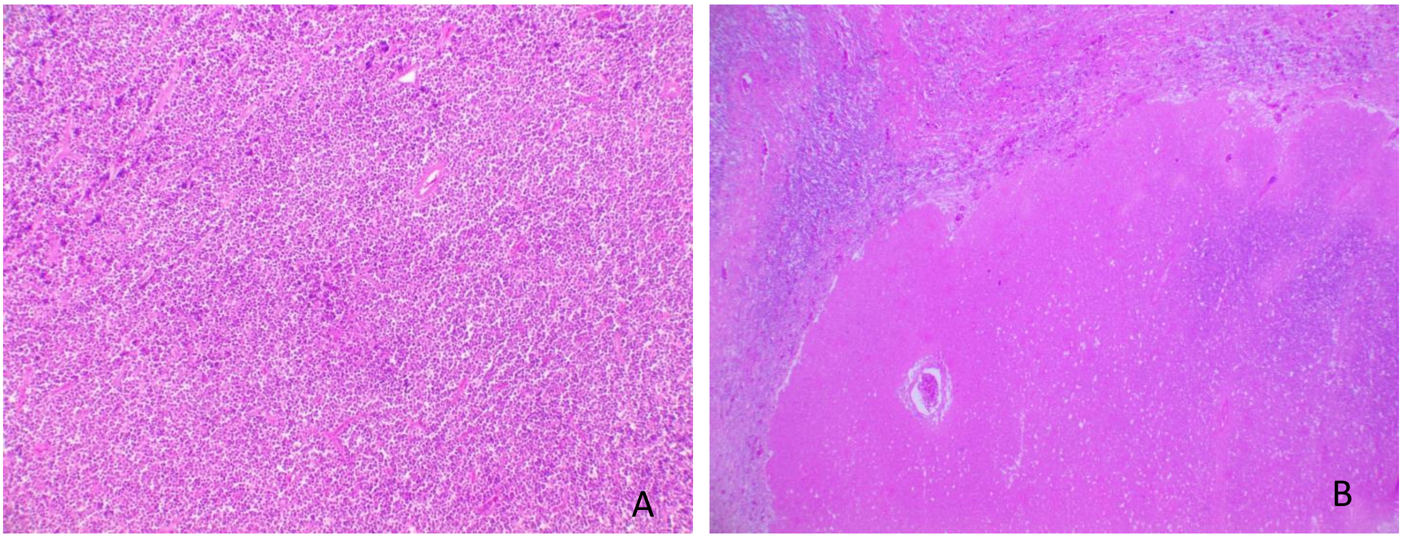

Figure S1. Lymphoma in the cervical nodule of the mallard duck. A) Biopsy fragment showing diffuse proliferation and mantle of lymphoblasts without identifiable tissue architecture. B) Nodule fragment from the necropsy exhibiting extensive necrosis (approximately 90%) with residual lymphoblast proliferation in the upper left portion. Stained with hematoxylin and eosin (HE).

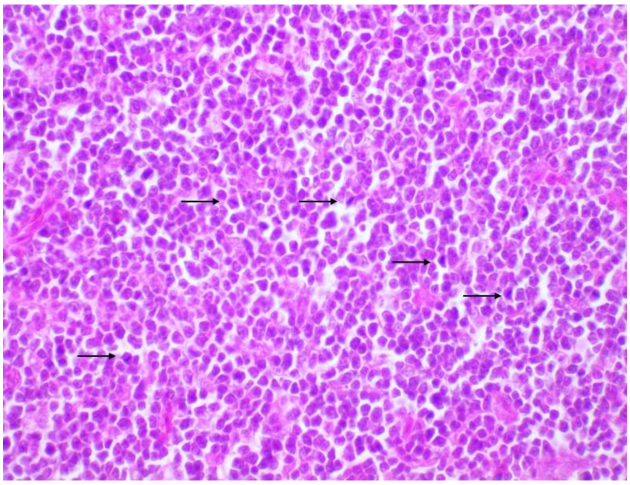

Figure S2. Lymphoma in the mallard duck. A) Proliferation of monomorphic lymphoblasts arranged in a mantle, supported by a discrete fibrovascular stroma. The cells exhibit a high nucleus-to-cytoplasm ratio, scant cytoplasm, and large nuclei with one or more prominent nucleoli. Frequent mitotic figures are observed (arrow). Stained with hematoxylin and eosin (HE).

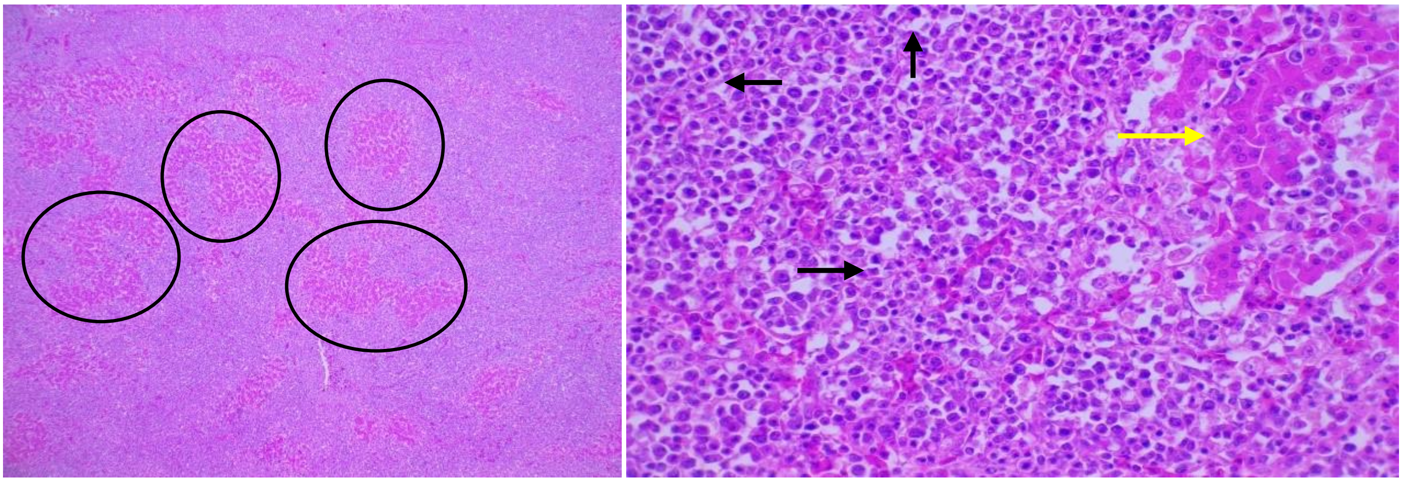

Figure S3. Lymphoma in the liver of chicken. A) Liver showing loss of normal tissue architecture, with extensive replacement of the parenchyma by coalescing areas of neoplastic lymphocyte proliferation. Residual hepatocyte cords are visible (circle). B) Proliferation of monomorphic lymphoblasts arranged in a sheet-like pattern. Frequent mitotic figures are noted (black arrow), along with remnants of hepatocyte cords (yellow arrow). Stained with hematoxylin and eosin (HE).

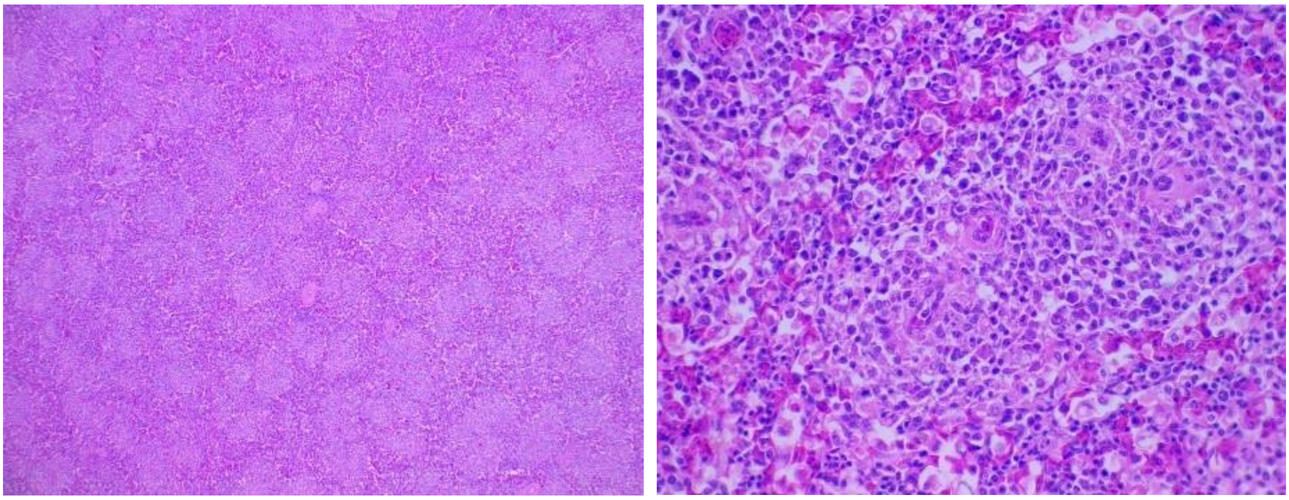

Figure S4. Lymphoma in the spleen of chicken. Nodular to diffuse proliferation of uniform lymphoid cells, resulting in the loss of normal splenic architecture. Stained with hematoxylin and eosin (HE).
